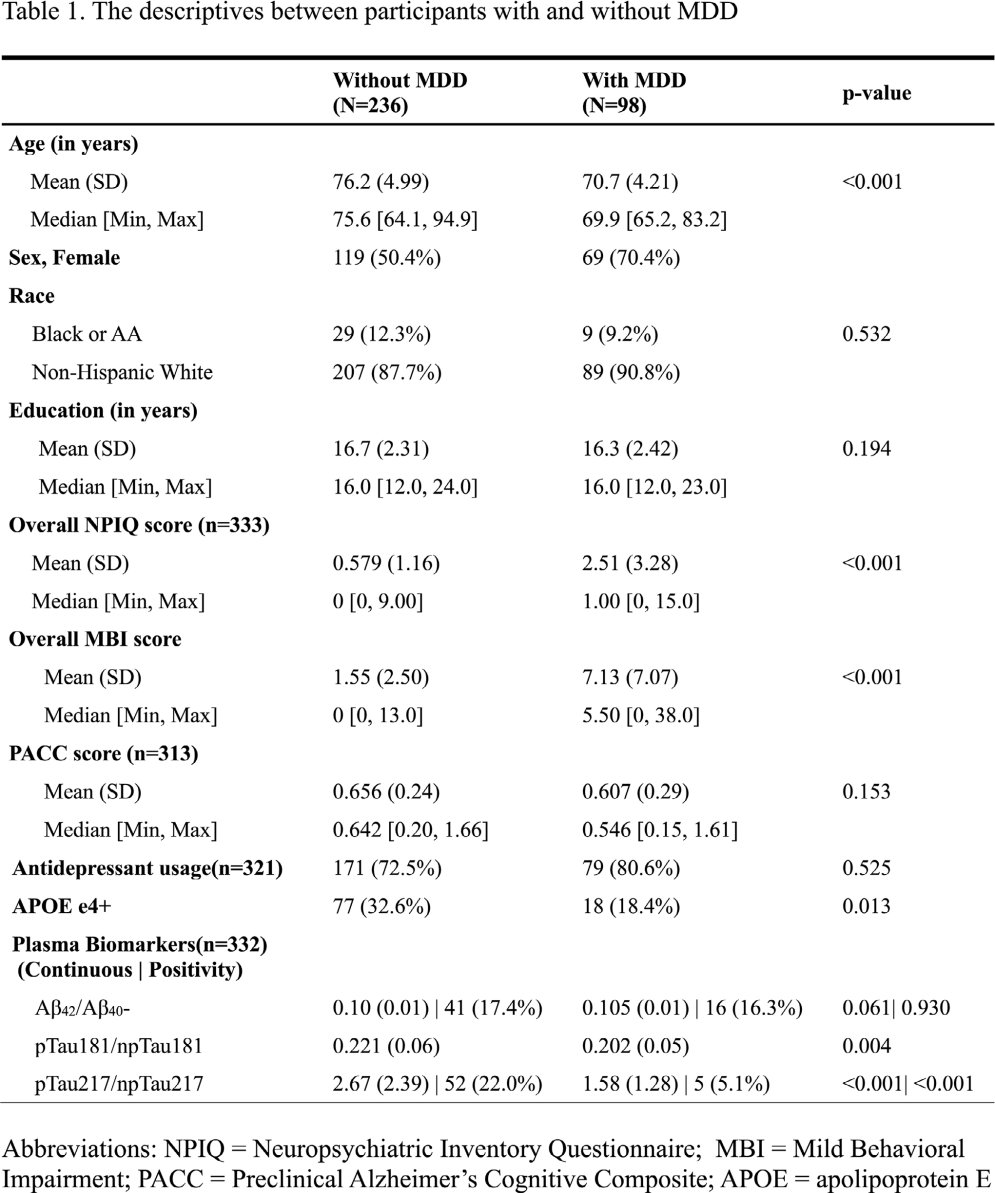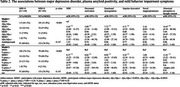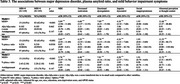# The associations between major depressive disorder, plasma biomarkers of Alzheimer's disease, and mild behavior impairment among older adults

**DOI:** 10.1002/alz70857_104024

**Published:** 2025-12-25

**Authors:** Yiqi Zhu, Jean‐Francois Trani, Ramkrishna Singh, Semere Bekena, Ganesh Babulal

**Affiliations:** ^1^ Washington University School of Medicine, Saint Louis, MO, USA; ^2^ Washington University, St. Louis, MO, USA; ^3^ Natiobal conservatory of Arts and Crafts, Paris, NA, France; ^4^ Washington University School of Medicine, St. Louis, MO, USA; ^5^ Washington University School of Medicine, St. Louis, MO, USA; ^6^ University of Johannesburg, Johannesburg, South Africa; ^7^ The George Washington University School of Medicine and Health Sciences, Washington, DC, USA

## Abstract

**Background:**

Biomarkers and neuropsychiatric symptoms (NPS) are two early indicators for identifying Alzheimer's disease (AD) and related dementias (ADRD). Major depressive disorder (MDD) shares NPS with ADRD, complicating diagnoses. Plasma biomarkers, while non‐invasive, require further investigation to confirm their accuracy and consistency. Although both plasma biomarkers and mild behavior impairment, a new measure of NPS, are independently associated with ADRD risk, their interrelationship, especially in individuals with MDD, needs further exploration. This study explored the cross‐sectional associations between plasma biomarkers and MBI among participants with and without an MDD diagnosis.

**Methods:**

Participants (*n* = 334) were community‐dwelling residents aged 65 and older and cognitively normal based on the Clinical Dementia Rating (CDR = 0). NPS were assessed using the MBI checklist. Plasma biomarkers including Aβ_42_/Aβ_40_, ptau_181_/nptau_181_, and ptau_217_/nptau_217_ were measured at C2N Diagnostics. MDD diagnoses were confirmed through medical records. Logistic regression models were used to evaluate the associations between MDD, plasma biomarkers, and MBI. The use of antidepressants and demographic variables were added to the models as control variables.

**Results:**

Among the 334 participants, 236 were non‐depressed, and 98 were from the MDD cohort. 188 (56.3%) were female. The average age was 74.6 years (SD = 5.39), and the average year of education was 16.6 (SD = 2.35). Of the participants, 38 (11.4%) self‐identified as Black or African American. There was no significant difference in race or education level between participants with and without an MDD diagnosis. Compared to participants with neither a MDD diagnosis nor Aβ_42_/Aβ_40_ positivity, participants with MDD diagnoses and negative Aβ_42_/Aβ_40_ were 5.42 (95% CI = 2.69–11.57) times more likely to endorse MBI symptoms. Participants with both MDD and Aβ_42_/Aβ_40_ positivity were 7.23 (95% CI = 1.87–47.81) times more likely to endorse MBI symptoms.

**Conclusion:**

MDD is associated with a worsening of MBI. Early identification and management of MDD may help reduce NPS severity. Differentiating ADRD from other neuropsychiatric conditions, such as MDD, remains a challenge. Further research is needed to clarify the distinct diagnostic and behavioral characteristics of ADRD and MDD.